# Attitudes Toward Aging among Widowed Women: Identifying Profiles and Associated Factors

**DOI:** 10.1093/geroni/igaf122.2492

**Published:** 2025-12-31

**Authors:** Yeonsoo Shin, Juhyeong Lee, Giyeon Kim

**Affiliations:** The University of Texas at Austin, Austin, Texas, United States; The University of British Columbia, Vancouver, British Columbia, Canada; Chung-Ang University, Seoul, Republic of Korea

## Abstract

Although previous research reports that widowed middle-aged and older adults’ attitudes toward aging are closely linked to their psychological well-being and health status, their distinct patterns of attitudes toward aging remain understudied. This study aims to identify patterns of attitudes toward aging among widowed middle-aged and older women and examine factors associated with these profiles. Using a nationally representative sample drawn from the 2020 and 2022 Health and Retirement Study (HRS), this study analyzes 1,223 widowed women, aged 48 to 99. The dataset includes responses to the Leave-Behind Questionnaire, which was distributed to half of the participants in 2020 and the other half in 2022. Attitudes toward aging were assessed using eight items on a six-point scale. Results from Latent Profile Analysis identified three attitudinal profiles based on attitudes toward aging, with higher scores indicating a more positive perception of aging: Low (36.7%), Middle (30.7%), and High (32.5%). Results from multinomial logistic regression showed that, compared to the Low group, older age (OR = 0.95, p<.001) and higher depressive symptoms (OR = 0.75, p<.001) were associated with lower odds of belonging to the High group. In contrast, those with better self-rated health (OR = 1.57, p<.001) were more likely to be in the High group. These findings highlight the role of self-rated health in fostering positive attitudes toward aging, while older age and depressive symptoms may be linked to more negative perceptions. These results suggest opportunities for interventions to support widowed women develop a more positive perspective on aging.

